# Scratchpads: a data-publishing framework to build, share and manage information on the diversity of life

**DOI:** 10.1186/1471-2105-10-S14-S6

**Published:** 2009-11-10

**Authors:** Vincent S Smith, Simon D Rycroft, Kehan T Harman, Ben Scott, David Roberts

**Affiliations:** 1 Natural History Museum, Cromwell Road, London, SW7 5BD, UK

## Abstract

**Background:**

Natural History science is characterised by a single immense goal (to document, describe and synthesise all facets pertaining to the diversity of life) that can only be addressed through a seemingly infinite series of smaller studies. The discipline's failure to meaningfully connect these small studies with natural history's goal has made it hard to demonstrate the value of natural history to a wider scientific community. Digital technologies provide the means to bridge this gap.

**Results:**

We describe the system architecture and template design of "Scratchpads", a data-publishing framework for groups of people to create their own social networks supporting natural history science. Scratchpads cater to the particular needs of individual research communities through a common database and system architecture. This is flexible and scalable enough to support multiple networks, each with its own choice of features, visual design, and constituent data. Our data model supports web services on standardised data elements that might be used by related initiatives such as GBIF and the Encyclopedia of Life. A Scratchpad allows users to organise data around user-defined or imported ontologies, including biological classifications. Automated semantic annotation and indexing is applied to all content, allowing users to navigate intuitively and curate diverse biological data, including content drawn from third party resources. A system of archiving citable pages allows stable referencing with unique identifiers and provides credit to contributors through normal citation processes.

**Conclusion:**

Our framework  currently serves more than 1,100 registered users across 100 sites, spanning academic, amateur and citizen-science audiences. These users have generated more than 130,000 nodes of content in the first two years of use. The template of our architecture may serve as a model to other research communities developing data publishing frameworks outside biodiversity research.

## Background

Taxonomic, systematic, and biodiversity studies (herein referred to as 'natural history') are data-intense sciences that draw information from many disciplines in order to build a coherent picture of the extent and trajectory of life on earth [1]. These data are essential to our discovery, understanding, and responsible use of the natural world [2]. Natural historians have traditionally relied on manual systems and techniques to gather, organise, and publish this information. This is collectively enshrined in scientific papers that form an archive spanning more than 250 years of published research. But as ever more synthetic and integrated accounts of the natural world are required to understand and mitigate threats to the environment, the failings of traditional publication methods for disseminating and using natural history research have become ever more apparent.

Traditional papers struggle to accommodate the high volume of data supporting accounts of natural history. In recent years this has been exacerbated by the rapid growth of semi-automated data gathering techniques producing large-scale datasets such as those incorporating genomic, phylogenetic, and image based components. The size and diversity of these datasets mean that they are at best marginalised to an electronic ghetto on publishers' websites. But all too often natural history data are simply never published [3]. The low impact of most natural history research, coupled with the high transaction costs associated with publication and access, mean that much (perhaps most) natural history data only exists tacitly and informally within expert networks of specialists (i.e. in the minds, notebooks and computers of the people generating the data). Such data are imperilled by a decline in the number of professional specialists engaged in these networks, such as that reported amongst biological taxonomists. Indeed, this had lead some to question the long-term viability of natural history as a professional scientific discipline [4]. Arguably natural history's salvation lies in better use (reuse) of the underlying data.

Meaningful forecasting and sustainable use of biotic resources requires large volumes of primary biodiversity data. However, this leaves informaticians with the challenge of integrating data from numerous, disparate natural history data providers, each with their own specific user communities, and diverse data types and sources, including taxonomic names and concepts, specimens in museum collections, scientific publications, genomic and phenotypic data, and images [5]. One approach is to data-mine existing publications in an attempt to reverse engineer scholarly publications into a database [6]. This approach is arguably the only option for legacy natural history data. But for new information, or that which has never been formally published, such reverse engineering should not be required so long as a technical, social and policy framework for data publication can be found. This paper is about the creation of a data-publishing framework for natural history.

Data publication is a new sort of scholarship that involves construction of large-scale datasets whose governance, organisation and use can be implemented using the web as a platform for generating, repurposing, and using data [7]. These systems offer the possibility of radical new ways of conducting scholarship and challenge some established ideas of how particular disciplines operate. To facilitate reuse and repurposing of data, these systems embrace 'Open Science' - the proposition of a model of communication inspired by the Free/Open Source software and Creative Commons movement [8]. A central theme of Open Science is to make clear accounts of the methodology, along with data and results extracted therein, freely available via the Web. This permits a massively distributed collaboration.

Design concerns relevant to data publication frameworks include the successful management of large scale distributed research programmes, how to support networks of independent researchers, the management of individual research careers, the development of new inter-disciplinary collaborations, and engagement with non-scholarly communities as both producers and consumers of research. These concerns are highly relevant to the challenges facing natural history scientists [4]. From a technical perspective these systems involve the development of tools for sharing natural history data, building and maintaining collaborations both formally and informally, and managing workflow and outputs.

A defining feature of data publication frameworks is that they primarily rely on social information flows, motivations and relations to organise the group. Individuals self-identify, mostly, for tasks, and through a variety of peer-review mechanisms contributions are recognised by the group and incorporated into what emerges as the collaborative output. A feature critical to their success is the ability of the framework to be broken down into discrete modules, capable of independent completion in relatively fine-grained increments. Because of this, people can contribute a little or a lot depending upon their motivations, such that some combination of 'true believers', occasional contributors, and people paid to participate can sustain a project.

In this paper we describe the system architecture and template design of "Scratchpads", a data-publishing framework for groups of people to create their own social networks supporting natural history science. This infrastructure is a combination of databases, network protocols and computational services that brings people, information and computational tools together to perform and publish natural history. Our goal was to build a system that could motivate individual researchers in the generation, management and dissemination of their own data for their own needs, while empowering a wider constituent of potential users who are free to repurpose this information for other uses.

## Implementation

### Design considerations and related work

Standard tools can be designed for the codification and dissemination of data and knowledge for communities with standard practices. But in cutting-edge disciplines, those spanning multiple specialities, or where standards are nascent and cannot be well defined, developing a common approach can be extremely difficult. Natural history science has all these challenging criteria. Natural history scientists work in fragmented, highly distributed and parochial communities, each with domain specific requirements and methodologies [9]. Their output is heterogeneous, high volume and typically of low impact, but with a citation half-life that may run into centuries. This output (e.g. species descriptions) broadly conform to a power law (long-tail) distribution where the least regularly accessed content accounts for more than half of the total and is proportionally more important than the smaller fraction of more regularly accessed (popular) content. Consequently a high level and flexible approach to developing the software and workflow is needed, covering broad subjects and themes in order to encourage adoption by a range of natural history scientists.

Fundamental to the design was the need to build a truly scalable and flexible data publishing framework accessible through a web browser that supports 1) large numbers of users as passive readers and active contributors; 2) editorial hierarchies serving individual and community needs; 3) the epistemological richness and diversity of all contributors; 4) flexible data models that can be modified or added by contributors; 5) automated integration of third party content; 6) automated semantic enrichment of contributed and third party content; 7) content workflows and curation tools; 8) content archival and citation; 9) content licensing and a conditions of use framework; 10) web services; and 11) ease of use.

Within the context of natural history science, some websites and services meet many of these needs across specific data types (e.g. TreeBASE for phylogenetic trees [10], Catalogue of Life for taxonomic names [11], GBIF for biological occurrence records [12]), or most of these data needs across narrow taxonomic domains (e.g., FishBase for fish [13], Avibase for birds [14], AmphibiaWeb for amphibians [15]). Arguably, though, none of these systems scale to the breadth of all taxonomic diversity for all natural history data types. Indeed most limit their scope in order to place some practical boundary on their development, and to simplify the process of establishing credibility within their chosen domain. Further, these systems all struggle to accommodate conflicting or alternative hypotheses about data. Natural history science is well known for its epistemological richness and diversity [16]. It is difficult, if not impossible, to find researchers completely agreeing with each other within and between domains (e.g. in the taxonomic classification or phylogenetic relations of particular taxa). Electronic systems that force contributors to adopt a single representation of a particular data set (e.g. a single taxonomic hierarchy for navigating data) risk disenfranchising potential contributors, often to the exclusion of their data and interpretations.

To address these design requirements and social challenges the Scratchpads consist of a loosely coupled platform for publishing natural history research that enables contributors to build, share, and manage data with minimal barriers beyond our highly generic design constraints. We accommodate epistemological diversity and the problem of establishing trust within natural history domains by enabling contributors to create independent sites whose purpose, destiny and brand rest in the hands of the contributing community. These communities are often well established and have a strong sense of purpose. Nevertheless, our software platform "Scratchpads" needed to establish credibility and trust across natural history science domains. This was achieved by basing the project at the Natural History Museum (NHM), London, and managing the project through the European Distributed Institute of Taxonomy (EDIT). The NHM has a well-established brand as a world leader in natural history research, while EDIT is a network of 28 leading European, North American and Russian institutions specialising in natural history. This structure was intended to minimise individual and institutional rivalries that might jeopardise the long-term sustainability of the project. Thus supporting our goal of creating an open community resource for natural history.

The unusual nature of this project demands agile development methodologies to promote frequent inspection and adaptation of the software in response to user needs [17]. Throughout this ongoing process we are mindful that our approach must be generic enough to scale to the widest constituent of possible users. Short development cycles and informal project management, sometimes spanning hours to generate several iterations of a feature in reciprocal response to user feedback, makes traditional documentation and planning difficult. However, our experience suggests this leads to software that better meets user needs, and fosters a community of developers and users that arguably builds a path toward long-term sustainability.

### Architecture and workflow

Rather than expand the taxonomic or content scope of an existing system supporting natural history science, we decided that a more generic solution would be required that could be tailored in a sustainable way toward the bespoke needs of natural history scientists. Content Management describes the set of processes and technologies that support the evolutionary life cycle of digital information. Content Management Systems (CMS) can provide generic informatics solutions for web publication of content created by individuals acting alone, or within organisations and research communities of almost any size. These tools aid in managing the development of software, collaborations, documents and websites. They are highly extensible and can be developed to support other research-specific activities such as handling large distributed datasets, data visualisation and analytical tools [18]. The emphasis of CMS tools on instant (web) publication and content maintenance, allows contributors to focus on content development, instead of administration.

Our decision to adapt an existing CMS for managing biodiversity data is a significant departure from the conventional approach employed within the biodiversity informatics community. Traditionally this involves developing customised database models, usually after extensive mapping and observation of the target user community, followed by the development of a bespoke software application that formalizes workflows and processes. A current high profile example of this approach is the Common Data Model (CDM) [19], which is in development by EDIT Workpackage 5 [20]. This is intended to provide a generic data model and service framework for bespoke biodiversity applications that are collectively referred to as the "CyberPlatform" [21]. Another high profile example is CATE (Creating A Taxonomic E-science) [22], which augments the CDM library and server with additional logic to present the data as web pages and in a workflow geared toward revisionary taxonomy. In our experience the challenge with these and similar bespoke biodiversity applications is threefold:

1. The data types, structures and workflows modelled within these systems typically do not capture the full gamut of data and practices engaged in by the wider user community. Consequently, take up amongst the potential pool of candidate users may be low beyond those surveyed, because the transaction costs for users engaging with systems that do not meet the full spectrum of their needs is too high. For example, biodiversity informatics applications often assume data held by users is more structured, and therefore more readily modelled within a database, or structured differently than it typically is. The effort (transaction cost) required by users to sufficiently structure (or restructure) their data is too high, relative to their perceived benefit from using the system.

2. The relationship between content (text, images, video, data etc) and context (layout presentation, branding, ownership, identity, audience etc) is crucial to understanding how and why users engage with information technology systems. This textography (sensu Swales [23,24]) is crucial to scholarly discourse, but is challenging to accommodate in biodiversity informatics systems that usually disarticulate the process of capturing content (e.g. the act of populating a database) from content presentation (e.g. a the display of a formatted database query on a webpage).

3. The heterogeneity of biodiversity data, coupled with multiple small, niche user communities, often with distinct needs and different audiences, requires highly bespoke informatics solutions. These are expensive and challenging to sustain, and usually lack a clear business model beyond intermittent cycles of grant funding.

Our reason for adopting a CMS as a platform rather than building a bespoke application was to directly address these challenges. The 'content' in CMS is typically loosely defined and can be accommodated in various ways, from highly unstructured 'pages' or nodes, through to highly structured normalized datasets. This provides the flexibility necessary to accommodate use cases that were not originally envisaged at the outset of the project. CMS minimize the distinction between the organization of content and its final presentation. This helps the content provider visualise how content will be presented to their audience without having to second-guess how an informatician will re-present content on their behalf. Finally, generic CMS systems are used extensively in many scholarly and non-scholarly settings and support a range of generic functionality that is required by all web-based applications regardless of the size or purpose of the userbase. Developer communities writing the underlying CMS software are completely independent and several orders of magnitude larger than the niche informatics communities working to support taxonomy and systematics. This gives generic CMS software much greater sustainability than bespoke biodiversity applications written by very small number of developers, which are supported by intermittent research grants. Building on top of a CMS removes the burden of having to develop generic functionality common to all applications (e.g. user management and content versioning) allowing developers to focus on specialised functionality that directly meets the needs of the target user community.

CMS are usually built on top of content management frameworks (CMF) and open source programming languages. Many CMS tools exist [25] but the top four based on a recent study [26] include one written in Python, Plone [27], founded on the Zope CMF; and three written in PHP: WordPress [28], a commonly used blogging platform; Joomla [29], widely recognised for its ease of use; and Drupal [30], commonly used in multi-user collaborative sites. For the Scratchpad project we selected Drupal because it offers a good balance between the sophistication and ease of use required for managing large and distributed user communities. Crucially Drupal met 6 of the 11 design criteria that were identified in the previous section. The variety of contributed Drupal modules (currently over 7,000), size of the userbase (including several Fortune 500 [31] companies, major universities and government agencies), and ready supply of developers thanks to the popularity of PHP, were all contributing factors in our decision. In principle the Scratchpad project could however, be replicated in any of the top four CMS.

The Scratchpad project was founded on Drupal version 5 in March 2007, although an ongoing transition to Drupal 6 means that as of June 2009 sites are being upgraded. The Scratchpad server's operating system is Red Hat Enterprise Linux (RHEL 5) running Apache 2.2 with PHP 5.1.6 and the database backend is MySQL 5.0.45. The Scratchpad project can, however, be run on any operating system, web server and database that supports PHP and Drupal. We are running a single Apache virtual host for all Scratchpad instances with Drupal handling domain names in a standard Drupal multisite configuration. This means that all sites share a common codebase but have their own database and database user, controlling data access on a site-by-site basis. User uploads are also segregated to independent folders to improve site security and facilitate site-by-site mobility, archival and backup. Drupal provides the foundations for basic web publication including the ability to register and maintain individual user accounts, administration menus, RSS-feeds, customisable layout, flexible account privileges, logging, a blogging system, and an Internet forum. The Scratchpads build on this through a combination of 42 modules provided by the Drupal community and 29 modules developed within the Scratchpad project (See Additional file 1: Modules for a complete list). The latter provide a suite of specifically adapted tools to support natural history scientists and their data.

New sites are initiated at the behest of a potential user, who registers interest and accepts responsibility for the new site, through a form on the Scratchpad website . Users supply their biographic details and information on their proposed use of the site, in addition to a Google Map API (Application Programming Interface) key that activates the Scratchpad geolocative features. Upon submission of the completed form, the site approval and creation process is initiated. Currently the Scratchpad project accepts all sites within the domain of natural history, even if their proposed subject overlaps that of a current site. Site creation is semi-automated and is controlled through a Drupal installation profile that specifies standardised Scratchpad settings, administrative users, modules, permissions, and establishes the site so that it is immediately accessible to the registrant. Registrants receive e-mail notification of their new site and are assigned the role of site maintainer, granting them administrative permissions that include the ability to assign new users as additional maintainers, editors or contributors. These roles control user actions through a cascading hierarchy of permissions. Contributors are restricted to authoring and editing their own content, editors can author and edit any content, while maintainers also have certain administrative privileges. Scratchpad project administrators have full administrative control from an account hidden to other users. A click through agreement ensures that each user agrees to a set of terms and conditions that outline their rights and responsibilities through use of the site . These terms are ultimately arbitrated by the Scratchpad administrators who reserve the right to review, refuse, monitor, edit or remove any content.

**Figure 1:**
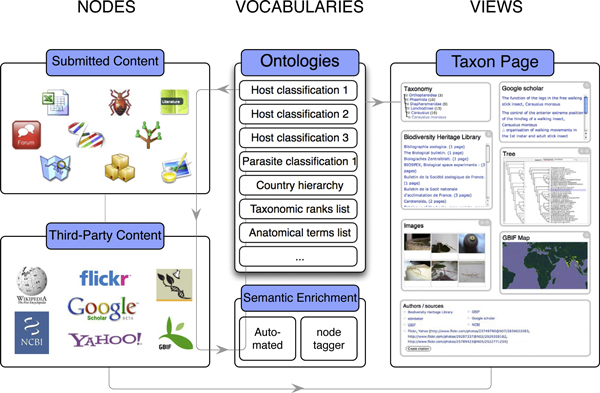
**Scratchpad workflow**. Users independently submit content in Drupal nodes and vocabularies of terms (e.g. biological classifications of taxon names) associated with content. Nodes are grouped into content types (e.g. images, DNA sequences, phylogenetic trees, GBIF maps etc), each of which has specific workflows for data entry and editing. Terms from a vocabulary (e.g. taxon names) common to nodes are used to semantically enrich (tag) content on submission. Tagged nodes can be represented in predefined views according to their content type, and are dynamically aggregated on term pages (e.g. taxon pages) that can be navigated through their vocabulary. Intuitive curation tools allow users to select which nodes to display on term pages. This model provides a highly generic solution to submitting, grouping, enriching, and navigating diverse data types.

Content is added to a Scratchpad through a flexible workflow (Figure 1) that is optimised if one or more ontologies (e.g. a biological taxonomic hierarchy) are imported into the site first. These ontologies provide a structure around which content can automatically be tagged (i.e. classified), and facilitate search and browse functionality. Scratchpad users do not have to follow this workflow when publishing content, although the absence of matching terms (e.g. taxon names) in an uploaded ontology will disable features that dynamically aggregate tagged content (e.g. Taxon pages), making this information harder to navigate.

### Vocabularies

Drupal supports the use of multiple restricted vocabularies as flat lists of terms, single hierarchies, or multiple hierarchies. We have adapted this system to support the import, export and management of biological classifications through a series of modules. They allow Drupal to support very large hierarchies of a potentially unlimited number of terms; improve the term editing and management interface; and provide the means to store additional term metadata. By default all hierarchical classifications are stored as parent-child relations within the site database. However, performance issues make this very inefficient for classifications with large numbers of terms. In instances where classifications contain more than one thousand terms, the hierarchy is additionally stored using a nested set algorithm [32]. This is implemented in a module  that has been tested with classifications exceeding two million terms without encountering notable performance degradation.

Drupal allows users to create multiple vocabularies that must be linked with appropriate content types before they can be used. This permits users to associate content with multiple, and potentially competing, classifications. Terms can be added to a vocabulary within a Scratchpad on a term-by-term basis through an editing interface, or en masse, either via a tab delimited text file that has been exported from a Spreadsheet template , or through a web service provided by the Encyclopedia of Life (EOL) project [33]. The latter enables users to import all child terms within a hierarchy from one of a selection of published biological classifications. This service supplies terms and associated metadata (authority, rank and synonymy) in an RDF representation of the Taxonomic Concept Schema [34].

Hierarchy management and term (taxon name) modification is supported for biological classification through an editor that enables intuitive (drag and drop) manipulation of terms and sections of hierarchies. This was developed in partnership with the Encyclopedia of Life project [33] and allows users to build new classifications using sections of classifications that have been previously created (i.e via drag and drop editing). The tool also allows users to associate term names with their protologue, i.e. the bibliographic reference of the original taxon description.

### Content types

Drupal stores content in nodes which are instances of content types, each of which may have bespoke workflows for data import, export, editing and visualization. Certain generic content types (e.g. pages, blog entries, fora) are defined within the core of Drupal. More specialized content types are defined through modules that are tailored to the particular demands of different data. The Scratchpads use a combination of contributed modules (i.e. those provided by members of the Drupal developers community) and modules written by members of the Scratchpad developers group. When a module developed within the Scratchpad project is sufficiently robust and of generic use to other Drupal users, it is released to the Drupal community via the Drupal website under an open source licence.

#### Bibliography

A contributed module  that allows users to manage and display lists of scholarly publications. As part of the Scratchpad project we use this as a stand alone content type in addition to integrating this into the workflow for managing bibliographic metadata associated with taxonomic names and specimens records. Features of the bibliography module include the ability to import reference lists in BibTeX, RIS, MARC, EndNote tagged and XML formats, and export lists in BibTeX, EndNote tagged and XML formats. The module allows users to format references in multiple styles and supports in-line citation of references. This function is currently one of the most popular features within the Scratchpads, supporting 37,204 nodes across 37 sites. Users can upload PDF files of articles to create discipline specific bibliographies relevant to natural history. Current Scratchpad bibliographies include examples on fossil insects  and fungus gnats .

#### Blog entry

A blog entry is a single post to an online diary or journal. This is a core content type within Drupal. Users can create multiple blogs within a site that are linked to terms within a restricted vocabulary. A good example containing multi-authored blog entries can be found on the Wallace Fund Website .

#### Character project

This node type allows users to build and manage a matrix of controlled, text or numeric characters associated with selected taxa. This matrix has the appearance of a spreadsheet, and can be used to build morphological or molecular datasets for phylogenetic analysis, identification keys and printed character lists or descriptions. On initiating a character project, users select taxa from one of any vocabulary present within their site using a hierarchical select list. Taxa may be hierarchically arranged within the matrix according to their classification, with an option for parent taxa to inherit the properties (character states) of their children, or arranged as a simple flat list. New characters are added to the character project at the behest of the user, who can choose between three character types. These are controlled characters, which limit character state choices to a restricted list provided by the user, e.g. molecular DNA bases; text characters which allow unrestricted textual input such as a verbose description of a particular morphological feature; and numeric characters, which allow whole integer or decimal input e.g. measurements. Characters can be added to character groups and can be reordered in a drag and drop fashion to facilitate the rapid creation and collection of character data. Users enter character states directly into the character grid, which are validated according to the type and controls specified when the character was created. Character projects use the SlickGrid jQuery plugin [35] that is incorporated into our character editor (nexus) module to provide the grid interface.

#### Countries map

This node type is used for displaying maps of the world highlighting selected countries. It is intended for use in displaying the presence or absence of taxa from particular countries, when more precise geolocative data is unavailable. Users select geographic regions from a list hierarchically organised around the TDWG geographic region ontology (levels 1-4) [36] to produce distribution maps of taxa. This module uses polygons corresponding to country outlines generated though a service provided by EDIT [37], which are overlayed onto a world base map presented in the Mercator projection.

#### Custom content types

Users can customise content types to their bespoke needs using the contributed Content Construction Kit (CCK) . The module is not a content type in itself, but allows users to create content types. CCK provides an intuitive interface for users to augment predefined content types (i.e. add new fields) or create new content types. Users first define a set of fields that become part of a table within the Scratchpad site database. Data can then be selectively imported into the table from a tab-delimited spreadsheet. This is done with the aid of an intuitive interface that guides users through the process of matching column headers from the spreadsheet with fields in the content type. Data can then be imported en masse, and visualised in user-defined presentations with the views and taxon page interface (see below). To date Scratchpad users have created custom content types for diverse data sets that could not have been anticipated by the Scratchpad development team, or supported because of its niche relevance to a particular subject domain. In many cases predefined standards for these obscure natural history data types do not exist. Examples include a checklist of cockroach cultures currently being held in captivity by members of the Blattoidea culture group  and descriptions of mosquito morphology (e.g. ).

#### Forum topic

A forum topic is the initial post to a new discussion thread within a forum and is a core content type within Drupal. The default profile of each Scratchpad includes a forum that users can customise. All users with login authentication can initiate and contribute to a forum. Fora can be linked to an e-mail account into which user can receive posted messages. An example of an active forum within the Scratchpads can be found on the Araceae Network Scratchpad (see ).

#### Group

Groups enable users to manage and control access to collections of content by adding content or other users to a group. This enables sub-communities to exist within a Scratchpad site, allowing site members to self-organise around public or private topics of interest, such as the production of scientific content for a research project. A group is created by a single group owner that has special permissions, including the ability to delete the group. Group subscribers communicate amongst themselves using the group home page as a focal point. Users can establish private groups that will not be displayed in the groups list, and groups may be selective, requiring users to be approved by the group administrator before becoming a member. Groups are defined through the Organic Groups contributed module .

#### Image

The upload and display of images is defined through a contributed module  that we have incorporated into a workflow through a second module . This supports the mass upload and display of images. Users can drag and drop collections of images in most formats including BMP, JPEG, TIFF, and PNG files, on to an open source applet [38] that will upload the pictures. Image thumbnails are dynamically created on upload. Users can select images on to which they can apply annotations en masse via the matrix editor (described below). All annotations are optional, and by default users can specify a title and gallery name from where the images will be accessible. Users can also apply keywords and details of preparation and imaging techniques. These are drawn from a restricted vocabulary defined within the Scratchpad that can be augmented by the user. For images of biological specimens users can optionally apply specimen and location information as defined by the Darwin Core standard [39]. Images can also be associated with publications through integration of this workflow with the bibliographic module. Licences specifying how images can be used can be applied through the Creative Commons module . Once uploaded, images and their annotations can be browsed through in an image gallery. This is a contributed module that allows users to group and organise collections of uploaded images. Galleries can be hierarchically arranged and include a weighting feature to control their placement on a page. Thumbnails of the first image within a gallery are dynamically created when an image is uploaded.

#### iSpecies Cache

This is an administrative content type that holds copies of third party content drawn from external services. It is used as part of the taxon page display where content is dynamically created around terms held in a site vocabulary (see below) and is hidden to non-administrative users. Many third party web services serving natural history data are fragile or slow to load. Caching this content reduces load time for users. The module is named in homage to Roderic D.M. Page's iSpecies website [40] that generates "on the fly" pages of species information.

#### Location (DwC 1.2.1)

A location record conforms to Darwin Core 1.2.1 fields [39] and can be used independently or in association with the specimen record content type. This is integrated within the workflow for annotating specimen records. Examples of Scratchpads that use this content type include sites on macrostomorph flatworms  and flies . The module allows users to specify a point location by clicking on a Google Map and dragging the location marker. The interface also supports the textual input of geolocative data matching the Darwin Core 1.2.1 standard, including information on elevation and depth.

#### Newsletter issue

This contributed module  publishes and sends newsletters to lists of subscribers. Newsletter issues are essentially e-mail messages that are stored within the site and associated with different newsletters through a list held by the taxonomy module. Users can edit this list to create new newsletters and users can subscribe to a newsletter within their user account page.

#### Page

A page is the simplest content type for creating and displaying information. Pages contain no predefined structure and are most appropriately used for content (i.e. text and images) that rarely change, such as an "About us" section of a website. The main field of the page can be optionally used with a WYSIWYG editor provided through the contributed WYSIWYG module . This provides an API that supports third party editors as plugins, simplifying the administrative process of adding and changing the editor. Currently we use the TinyMCE editor [41] across the Scratchpads.

#### Phylogenetic tree

This module is based on Roderic D.M. Page's tree viewing widget (TVWidget) that displays very large phylogenetic trees to a constrained and predefined size. The widget [42] has been wrapped within a Drupal module called Tree and allows users to paste or upload a Newick tree description or Nexus file containing a Newick formatted phylogeny. The module creates a page that displays the widget. Examples of very large phylogenies displayed in Scratchpads through this module include trees on Dung beetles  and termites .

#### Poll

Polls are questions with a set of limited responses. Authorised users can create a poll and invite other users to vote on the responses. Once created, polls automatically provide a running count of the number of votes received for each response. This is a Drupal core content type.

#### Specimen (DwC 1.2.1)

Specimen records are based on the Darwin Core version 1.2.1 fields [39]. This content type allows users to record biological specimens through a tabbed workflow and is linked with the location Darwin Core content type. An example of a site that uses this facility is a Scratchpad on Freeloader flies .

### Nodes

All content is stored in Drupal nodes which minimally have a unique URL, title, creator and last edited/created times, in addition to any supplementary fields defined by the content type. Each node is given a numeric identifier that is addressable within the database and forms part of the URL. This identifier always links to the most recent version of a node if its content is modified. However, all previous versions of content are stored and can be accessed through a permanent URL. A URL alias is created from the title of the node that is supplied by the author. This alphanumeric alias is usually more intuitive than the numeric node identifier and can be customised by authorised users. However, the numeric node identifier cannot be changed and is persistent and unique within a site. When appended to the sites domain name this acts as a Globally Unique Identifier (GUID) that addresses node content. Optionally, each node can be tagged with one or more terms from a vocabulary allowing content to be searched and aggregated through these terms. At the base of every node a range of options are presented to the user that include menu settings (to attach nodes to a menu block), publishing options (to publish or unpublish a node, promote it to the sites front page and allow it to sit at the top of a list), comment settings (to enable or disable comments) and file attachments (to attach one or more files to the node).

All Drupal nodes can be translated through the contributed internationalisation package. This consists of a collection of modules that provide a translation interface for the creation of comprehensive multilingual sites including node content, taxonomies and menu items. Working in conjunction with browser language detection this will redirect users to content displayed in their preferred translation and includes a block for language selection. At present only one site on stick insects - the Phasmid Study Group  has made use of this facility.

### Semantic enrichment (tagging)

New or modified nodes are automatically searched upon submission to identify terms that match those present in any vocabularies associated with the node content type. If one or more terms match, the user is presented with an interface that enables them to select which term/s to associate with the node. These features are provided by the autotag module  that supports terms from multiple independent vocabularies, even if the term name is common to two or more vocabularies. Terms located in a node are pre-selected in the interface to enable fast and efficient tagging. The interface also facilitates tagging with terms that are not present within the node. Term name auto-completion, which includes references to the source vocabulary (i.e. classification), speeds up the input of additional term name tags.

### Blocks and views

Blocks and views are a means to create boxes of related/grouped data that can be built to create aggregations of content from multiple node types. Blocks are normally used in the left and/or right sidebar(s) of a site and are a core feature of the Drupal CMS. Views is a contributed module that acts as a powerful query builder. This allows users to fetch and present highly customised lists, tables and other visualisations. Detailed descriptions of blocks and views can be found in the Drupal handbook . Here we focus on how we have used these features within the Scratchpad project.

The Scratchpad profile specifies a series of blocks for the left and right sidebars including a ClustrMap [43] that shows the location of site visitors; a tabbed search block that allows users to switch between free text searches of the entire site, and filtered searches based on an auto completed list of taxon names stored within the site vocabulary; an 'about this site' block providing brief background information supplied during the Scratchpad signup process; and a user log-on block. Once logged in the log-on block is replaced with an administrative menu and a 'Create Content' block providing direct links to major site features.

The default data views created by the Scratchpad profile on creation of a site include views for each content type including data from third party sources. These are customised to the particular needs of each data source. For example, bibliographic data are displayed in a table that can be sorted by author, year and publication title; images are displayed as a list of thumbnails; Wikipedia content is displayed as the first block of text before the first header; and GBIF occurrence maps are simply embedded within the view. Users can also create Google Map views displaying geolocative data with the GMaps module. This allows nodes containing georeferenced data to be filtered and plotted on a Google Map. By default Google Maps are created for all nodes with geolocative information, and all users who have specified their location in their user profile.

A view of nodes and their associated fields is dynamically created for each content type (including user defined CCK content types) and works in conjunction with a grid matrix editor we have created for spreadsheet-like data entry. This allows users to edit data en masse, where each row represents a node (web page) and each column represents a field in the content type. Data in each field is validated according to the same controls that would be present if it were being edited within a single node. The matrix editor allows users to rapidly edit large datasets in an intuitive spreadsheet like environment.

### Taxon pages

Taxonomic names provide a central link between diverse items of information about an organism. Given an organism's scientific name a wide range of data can be drawn together, including content from third party databases that have a suitable API. Scratchpad taxon pages are our attempt to allow users to dynamically construct and curate pages of information (e.g. phenotypic, genomic, images, specimens, geographic distribution) about any taxon regardless of the physical location of the source data. These pages can include information contained within a Scratchpad and data drawn from selected third party resources.

Taxon pages are built around the biological classifications that users have created within their sites, and can be intuitively navigated through the taxon hierarchy block, created with the TinyTax module . Because content may be inappropriately tagged with a taxon name, especially if it is drawn from a third party source, taxon pages include curation tools allowing users to select which content to display, and how this is arranged on a page. This is achieved through an intuitive drag-and-drop interface that supports the curation of the major categories of information to be displayed, and the detailed content within these categories. By default, content is dynamically aggregated on a taxon page in an order that represents the most recent information for any given category. Users can fix content on a taxon page using simple view controls that hide inappropriate content and force the display of particularly pertinent information that is likely to remain relevant. For example, users may select holotype and paratype specimen records to be displayed first, followed by a list of the most recently collected specimens. These controls operate on data regardless of whether content is drawn locally or sourced from a third party, allowing taxon pages to display a combination of fixed and recent information as selected by the curator.

An 'on-demand' citation feature allows users to create permanent snapshots of an entire taxon page, whose content will never change and will reside at a permanent URL. Buttons to request the creation of a citable version are placed at the bottom of each taxon page. When pressed these display a copy of the citation information including the URL and a Chicago style bibliographic reference to the archived taxon page. An option to send the citation data to the user as an e-mail is also presented. This citation feature is available to users as a Drupal block, making on-demand citation possible for all Scratchpad content on any node.

Taxon pages are built through a combination of blocks and views that are predefined by Scratchpad modules. Users can create new blocks and views of data that are available to the taxon pages, allowing users to customise this feature to their bespoke needs. Programmatically, taxon pages are defined through a series of modules that facilitate the sorting, caching and citation of content. In Drupal 6 sites these are brought together through the "Mado" module  that integrates the required modules and lays out the taxon page. This replaces our implementation of the Panels module  used in Drupal 5 sites.

### Third-party content

The international scope and interdisciplinary content of natural history means that most natural history data are presently distributed in thousands of public and private databases worldwide. Given a shared identifier and a suitable API, information from these databases can be integrated into taxon pages within a Scratchpad. These pages rely on taxon names as a shared identifier. However, the instability of taxon names (e.g. due to synonymy) coupled with the fact that they are not globally unique (e.g. due to homonymy) mean that taxon names do not always refer to the same entity. We rely on the taxon page curation tools available to users to address this problem.

There is no sustainable way of addressing the lack of an API for most natural history databases. Consequently we focus our efforts on the major data providers that, because of their size, scope and/or quality, have well specified API's and have become part of the scholarly communication process for natural history scientists. A list of current third party data providers accessible to Scratchpad users is given in Table 1. Adding new services requires an API that takes a taxon name as the search term. The return is processed and put into a list of results that is passed to the iSpecies module. The latter displays the results within a Drupal view which is processed within a bespoke module for each service. New services can be quickly and easily added given a data source with an appropriate API.

**Table 1:** Third party services used by the Scratchpads. Third party services integrated into the Scratchpad taxon pages. Modules are held in the Scratchpad SVN repository at: .

**Module Name**	**Description and API**
bhl	Searches the Biodiversity Heritage Library for printed pages held within their archives that have a reference to a specific taxon name.
	API:
flickr	Searching the Flickr image database for pictures that have taxon name metadata associated with them.
	API:
gbifmap	Displays maps of the world that geolocate biological occurrence records from the GBIF database.
	API:
morphbank	Searching the morphbank image database for pictures that have taxon name metadata associated with them.
	API:
ncbi	Searches the NCBI database for nucleotide sequences, protein sequences and related links.
	API:
wikipedia	Displays the initial section of a Wikipedia article for the taxon name, if the page exists.
	API:
yahooimages	Similar to Flickr, but for Yahoo! Images.
	API:

### Services

We provide a limited range of services on Scratchpad data. At present these are restricted to specimen and bibliographic data. However, Scratchpad users can create bespoke views of their data in XML format that can be accessed by others. This is achieved with the views module by specifying XML output and can be created by any user with appropriate permissions (currently site editors, maintainers and the Scratchpad administrators).

A service on specimen data is provided by TapirLink software [44] external to the Scratchpads. TapirLink uses each set of Scratchpad database specimen records as a data source. These data fit the DarwinCore v1.2.1 standard [39] allowing TapirLink to serve denormalized specimen data to interested partied including GBIF [12]. At present users have the option of having this service switched on.

Bibliographic data are currently available from the Scratchpads in BibTeX or Endnote format. BibTeX data is currently harvested by Falx [45], a bibliographic data harvester that is part of ViTaL (the Virtual Taxonomic Library), that aggregates bibliographic data from various sources including the Scratchpads to provide an integrated search interface for taxonomic literature of relevance to natural history research scientists (e.g. ). We also expose bibliographic data using the OAI-PMH (Open Archives Initiative Protocol for Metadata Harvesting) module [46]. This provides an OAI-PMH version 2 interface to bibliographic data held by the bibliography module within any Scratchpad. We plan to evaluate the use of OAI-PMH as a more generic solution for serving all data from the Scratchpads in due course.

### Support

The Scratchpad project is constantly evolving in response to users needs, under the programming mantra "release early, release often". Through regular user feedback, features undergo several revisions in a very short space of time. This pace of change makes traditional documentation (e.g. user manuals) difficult. The energy involved in producing traditional documentation often exceeds the likely benefit to users, since a described feature is often out of date before the majority of users realised the feature was present. To address this challenge we needed a modular user guide that could be quickly revised to reflect recent development. This was done using an e-Book within the Scratchpad website that contains a series of help screencasts outlining the functions and day-to-day operation of the site. These screencasts can be constructed quickly and slotted into the existing e-Book structure. To date almost 100 screencasts amounting to more than 8 hours worth of video are available to users (see ). In addition, we have constructed a support forum , FAQ list  and Sandbox  that rebuilds itself every hour, allowing users to explore the functionality of the Scratchpad project. If all else fails, users can e-mail us for assistance at support@scratchpads.eu.

### Administration

Administrative tasks within the Scratchpad can be accessed through the Drupal Administration page. By default users are presented with a simplified version of this page that hides most of the advanced or less well used administrative functions. The majority of Scratchpad users do not need access to the administrative components of the site, and many of these features are restricted to site maintainers. Here we will only consider Drupal administrative features that we have customised for the Scratchpads.

Drupal supports OpenID [47] and we have created our own OpenID identity provider  that allows us to provide users with OpenID identities. Our use of OpenID enables users to logon to multiple Scratchpads and other OpenID enabled websites with a single username and password. Users can specify OpenIDs from any identity providers in their user profile. From our implementation of Drupal version 6 we have customised the Scratchpad user profile to include a default set of fields that include the user's academic affiliation and domain of taxonomic expertise. These fields can be further customised by site maintainers to include additional fields relevant to the bespoke needs of the site.

Natural history researchers generally rely on social, and not financial motivations to contribute content. Consequently customised branding to establish group identity is important to the success of many sites. Drupal's core features support branding, allowing authorized users to customise the site title, slogan and mission statement, in addition to uploading a logo and adjusting the theme that controls the sites colour scheme and page layout. Drupal themes are usually implemented with Cascading Style Sheets (CSS) and a wide range of themes can be downloaded from the Drupal website . Within the Scratchpad project these functions are restricted to site maintainers, and accessed through the site information and theme pages within the advanced administration section of a site. By default, new Scratchpads are created with the multi-column, fluid width Garland theme that can be re-coloured using an intuitive colour picker. Maintainers can switch between a selection of compatible themes with a range of layouts and styles we have pre-selected for the Scratchpads. However, users cannot upload additional themes to their site. In practice most Scratchpad users accept the default layout settings and prioritise content creation over refined issues of site design and navigation. Consequently non-members may consider that many Scratchpads look inelegant or are difficult to navigate. These design and content-building aspects are part of the process by which members establish their sense of identity and community: a similar phenomena can be seen on many social networking websites e.g. MySpace[48].

Customised themes and workflows could be developed to promote good design and site navigation, while retaining the high level of flexibility needed by users to establish a sense of community identity. At present the only design restriction that is pragmatically enforced is the presence of a small footer, forced at the base of every Scratchpad page. This contains logos for EDIT, the Scratchpads, Creative Commons, and the Drupal CMS, each of which link to their respective projects with the exception of the Creative Commons logo. The latter currently links through to version three of an unported (i.e. international) Creative Commons license [49] that defines how content on the node can be used. By default this license permits non-commercial use by attribution, with the proviso that subsequent use of the content is covered by the same Creative Commons license.

The Scratchpad server is backed up using IBM's Tivoli Storage Manager [50], which archives the last seven versions of any file contained within the system. These archives are stored on tape, and are collected during weekdays and stored off site outside London. Site maintainers can individually backup their database and site content on demand through a backup module we have created. This could be used to re-establish the Scratchpad on another server or extract and repurpose the data.

## Results

The Scratchpads currently (June 09) support 1,262 registered users who have created 162,753 nodes of content across 100 different sites. Of these users, approximately 5% log in every week across one third of all sites. This rises to 13% of users per month across 50% of sites. A breakdown of statistics on registered Scratchpad users is available at . This list is dynamically updated every day. The biographic profiles submitted by registered users does not oblige them to identify their country of origin, but a crude analysis of domains from registered users' e-mail addresses suggests they reside in more than 50 countries worldwide.

Site visitors are recorded on our servers and through a Google Analytics account [51]. Scratchpad sites  breakdown into five major categories based on taxa, regions, societies, e-Journals/books, and services. However, many sites have overlapping purposes or change in scope over time, making a precise site classification difficult. The majority of sites are taxon focused, serving communities of natural history researchers specialising in particular taxonomic groups. Some of these communities are very large. Nanotax for example  is a well-developed site with 132 registered users working on calcareous nannofossils. Similar examples include sites on Fungus Gnats  and Aroid plants . Modest numbers of users (10-20) are more typical, such as a site on freeloader flies . In other cases a well-developed site may have a single active registered user (e.g. ). Geographically defined Scratchpads includes sites on the Spermacoce from Australia  and the Malesian Moss Handbook . A Scratchpad also hosts the Journal of the European Mosquito Control Association (European Mosquito Bulletin, see ) and the journal Phasmid Studies . Service sites provide facilities and tools that are not usually taxon-specific but are needed by natural historians. An example is the World Catalogue of Common Names  that aims to serve as a repository for common (or vernacular names) for any species or species group in any language. While most sites keep the majority of content public, some like the Howard and Moore Checklist of the Birds of the World  keep almost all content private. Often there is little correlation between the size of a site and the quality of its content. Some sites with the most nodes have only a handful of registered users, while some high member sites have relatively little content.

On the Scratchpad homepage we use a basic set of site statistics to encourage and motivate contribution. Sites with the greatest number of nodes, users and views are placed more prominently on the Scratchpad sites list, encouraging competition between site maintainers that are keen to promote their work. Within the Scratchpads a predefined block allow maintainers to place a badge containing basic usage statistic (users, views and nodes) on their site. Some site users take a keen interest in their site usage statistics, to the point of using personal Google Analytics accounts [51].

### Current and future developments

**Figure 2:**
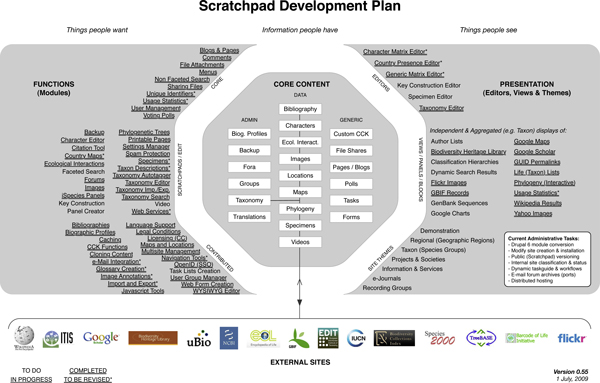
**Scratchpad development plans**. A crude outline of the Scratchpad functional development plan. Entries not underlined are planned, those with a single underline are in progress, those doubly underlined are complete, and those with an asterisk are complete but will be revised.

The path to sustaining the Scratchpads lies in distributing the hosting and software development activities across multiple institutions, and in embedding the software into the work practices of researchers. Our future development plans are focused on these goals. Specifically we are developing partnerships with other institutions and organisations that have the technical capacity to host and mirror instances of the Scratchpad server. We have an agreement with the Encyclopedia of Life "LifeDesk" project [52], which uses the same underlying software and approach as the Scratchpads, in an effort to share development activities. Experimental Scratchpad servers have been established at New York Botanic Gardens and Chicago Field Museum. Several European institutions have expressed an interest in hosting and developing sites. Our aim is to establish a foundation of partner institutions and organizations that will cover the costs of mirroring and hosting Scratchpads worldwide, and establish a social framework needed to coordinate and manage the project. These benefits will be largely invisible to users, except in the context of providing a more stable and reliable service. To more directly address user needs we also plan a suite of functional developments focusing on data capture, visualization and analytical tools that enable the Scratchpads to better fit within the work processes of taxonomists, systematists and the users of these data. Coupled with these are enhancements to facilitate greater mobilization of data held within Scratchpad sites. Space precludes elaboration of these tasks, however they are broadly outlined in Figure 2.

## Discussion

The Scratchpads as they currently stand act like an archipelago of islands, each representing a natural history domain that is internally consistent but largely isolated from the others. Services built into the Scratchpads achieve some limited level of interoperability, drawing categories of data in from various third parties and serving data on a handful of common data types. Scratchpads are also helping to deliver an enhanced level of social cohesion amongst natural history scientists, acting as a nexus for communities. But to achieve a future in which information from different disciplines is truly interoperable, something more is required.

Natural history scientists might coordinate their efforts more tightly, perhaps through a top down management approach. International organisations like GBIF [12], TDWG [53], and the Consortium for the Barcode of Life [54] are helping to deliver this. However, in a science noted for its epistemological diversity, top down efforts are hard to deliver to hands-on scientists. Technologies that are tolerant of diverse data models and can accommodate the ontological richness of natural history are more likely to succeed. Semantic web activity [55] offers this prospect, although the low uptake of semantic software suggests semantic web technologies are too nascent to deliver on this promise just yet [56].

Arguably in the short term, a third path to a more effective cyber infrastructure for natural history is more attainable. Using current technologies to build systems that encourage the deposition of datasets and facilitate community annotation and collaboration, we can gain many of the benefits envisioned for more sophisticated systems. This approach leverages the human capacity to make sense of noisy and contradictory natural history data, while accommodating machine methods like the automated tagging of content. Tools like the Scratchpads are not a definitive solution to the problem integrating natural history data. Rather, as an enabler of digital data capture and collaborative working, usage of the Scratchpads is a step toward a less fragmented and more integrated global natural history.

## Conclusion

The ordering of data across natural history disciplines is not simply a question of finding a commonly accepted database model or standard for representing biodiversity data. A more generic solution that supports the ontological richness and diversity of natural history is required. Scratchpad software is our attempt to meet this need for communities of natural history scientists where geographic constraints or limited access to data would otherwise impede progress. Scratchpads blend social, technical and policy developments into a common platform supporting natural history researchers in the creation and reuse of data. This data publishing framework makes it easy for groups of natural historians to self assemble around a common interest, and for individuals to contribute to group effort without formal management. In a biodiverse world we need to be able to manipulate and annotate ontologically diverse data. Scratchpads provide a framework to support this data diversity, and encourage metadata practices that support global efforts towards a more integrated approach to natural history.

## Availability and requirements

**Project name**: Scratchpads

**Project home page**: 

**Operating system(s)**: Platform independent (Web application)

**Programming language**: PHP

**Other requirements**: none

**License**: Web application is freely accessible for all users. Source code is available under GNU General Public License version 2.

**Any restrictions to use by non-academics**: none

## Competing interests

The authors declare that they have no competing interests.

## Authors' contributions

VSS designs and leads the project, and provides the biological and sociological insight that defines the Scratchpad program of work. This includes designing and testing the Human-Computer-Interaction (HCI) aspects of component modules and authoring most of the text and multimedia content on the Scratchpad project website and within project modules. SDR leads all aspects of the technical development, writing and integrating the package of software and providing the system administration. SDR also manages the additional technical developers including BS who developed the initial version of the Scratchpad taxon pages, and KTH who provides selected testing and user support. DR provides overall coordination of the Scratchpad activities within the EDIT program of work and handles project administration. DR also tests modules and is the maintainer for a scratchpad on ciliated protozoa . VSS wrote the manuscript with contributions from SDR and DR. Other authors provided editorial comments and approved the final draft.

## Supplementary Material

Additional file 1 Contributed (C) and Scratchpad written (S) modules used within the Scratchpad project. The classification module (EOL) has been developed with the Encyclopedia of Life's "LifeDesk" project. Contributed modules can be found at  [Project Short Name]. The Scratchpad modules and the EOL classification module can be found at  [Project Short Name]. Selected Scratchpad modules have also been published on the Drupal project website when we consider them to be of use to the broader Drupal community. Click here for file
