# Supplementary material for: Scratchpads: a data-publishing framework to build, share and manage information on the diversity of life
Source: BMC Bioinformatics. 2009 Nov 10;10(Suppl 14):S6. doi: 10.1186/1471-2105-10-S14-S6 (PMC2775152; doi:10.1186/1471-2105-10-S14-S6)
Supplement: Additional file 1 — Contributed (C) and Scratchpad written (S) modules used within the Scratchpad project. The classification module (EOL) has been developed with the Encyclopedia of Life's "LifeDesk" project. Contributed modules can be found at [Project Short Name]. The Scratchpad modules and the EOL classification module can be found at [Project Short Name]. Selected Scratchpad modules have also been published on the Drupal project website when we consider them to be of use to the broader Drupal community. [file 1471-2105-10-S14-S6-S1.pdf]

Additional File - Modules

Contributed (C) and Scratchpad written (S) modules used within the Scratchpad project. The classification module (EOL) has been developed with the Encyclopedia of Life's "LifeDesk" project. Contributed modules can be found at http://drupal.org/project/[Project Short Name]. The Scratchpad modules and the EOL classification module can be found at http://svn.scratchpads.eu/viewvc/scratchpads/branches/drupal6/sites/all/modules/[Project Short Name]. Selected Scratchpad modules have also been published on the Drupal project website when we consider them to be of use to the broader Drupal community.

Source	Project Short Name	Project Description
C	advanced_help	Provides an interface to the improved module documentation.
S	ahah_action	Provides a Norwegian pop band.
C	auto_nodetitle	Automatically gives nodes of specific content types a title.
S	autotag	Automatically associates nodes with terms within the node.
S	backup	Enables a site administrator to download a dump of the site's database, and all uploaded files.
S	batax	Alters the taxonomy input form to an autocomplete field, rather than a drop down box.
C	biblio	Provides the biblio content type for adding of references to a Scratchpad.
C	boost	Caches Scratchpad content to reduce server load.
C	cck	Allows for the creation of custom content types.
C	checkbox_validate	Required by the Legal module to enforce validation of required checkbox fields.
S	citation	Provides a block that enables users to create a permanent copy of the current page.
EOL	classification	Written by EOL, this improves the management of taxonomic classifications.
S	classification_biblio	Allows a taxonomy term to be associated with a biblio node.
S	classification_scratchpads	Adjusts the Classification module so that it works with the Scratchpad system.
C	contemplate	Allows administrators to adjust the way content types are displayed.
S	countriesmap	Provides a content type for displaying world maps with highlighted countries.
C	creativecommons_lite	Enables content creators to associate CC licences with a node.
S	darwincore	Provides the Darwincore specimen and location content types.
C	date	Provides additional fields for the content type module allowing users to enter dates.
S	fixperms	Fixes permissions associated with content types created by the node module.
C	fotonotes	Allows a user to add notes directly to an image on a Scratchpad.
C	globalredirect	Ensures that each node is displayed at one URL, redirecting others that can access the node.
C	gmap	Provides integration with Google Maps.
S	hashcash	Blocks spam content based on the Hashcash algorithm
C	i18n	Provides the translation and internationalization interface.
C	image	Provides the Image content type, and image galleries.
S	imagex	Enables multiple images to be uploaded to a site by dragging them onto a page.
C	imce	Provides an intuitive interface for uploading files to embed within text areas.
C	imce_wysiwyg	Links the IMCE module with the WYSIWYG module.
S	ispecies	Provides the iSpecies views used on Taxon pages.
C	jquery_ui	Extends the jQuery library.
C	jquery_update	Extends the jQuery library.
C	jstools	Provides collapsible menus and Javascript tabs.
S	leftandright	Improves handling of very large taxonomies, enabling them to handle in excess of 2 million terms.
C	legal	Enforces confirmation of the terms and conditions of use document during user registration.
C	lightbox2	Provides the image popups in image galleries and Taxon pages.
C	location	Allows a node to be associated with a location.
S	lowername	Improves searching of taxonomy terms by autotag, taxtab, and related modules.
S	mado	Provides the block layout of Taxon pages.
S	matrix_editor	Provides a grid interface for creating editable views, and also hooks into the Nexus module.
C	messaging	Provies the API for other modules to send emails and messages to users.
C	mollom	Protects a site from spam by checking content using the http://www.mollom.org service
S	nagger	Enables a user to restrict what roles can and cannot see in a node.
S	nexus	Provides the character data editor.
C	node_clone	Allows a user to quickly clone an existing node for subsequent editing.
C	node_import	Supported node import from Tab or Comma delimited files.
S	node_term_edit	Allows automated node association with terms by appending ?tid=[TERM ID] to the node edit page.
C	notifications	Notifies users when content is added to a site.
C	og	Provides a way of grouping content within a site that can only be accessed by group members.
C	pathauto	Generates URL aliases for a node.
C	path_redirect	Redirects URLs from old url aiases to new ones.
C	print	Provides a link to print friendly versions of most pages.
C	quote	Enables users to quote other comments when replying in forums.
C	robotstxt	Allows the robots.txt file to be edited.
C	roleassign	Allows Scratchpad maintainers to assign roles to new users.
S	scratchpadify	Provides some of the adjustments common to all Scratchpads.
C	search_files	Enables the contents of attached files to be indexed for search terms.
C	simplenews	Provides the Newsletter issue content type.
S	spm	Provides a Species Profile Model content type.
S	tablesorter	Enables a table created by the views module to be sorted using Javascript.
S	taxonomy_tree	Provides a hierarchical checkbox list for selecting taxonomy terms.
S	taxtab	Adjusts the search form so it will autocomplete with vocabulary terms.
C	thickbox	Provides popup boxes that are used by the Citation module.

S	tinytax	Provides a sidebar block with a vocabulary to facilitate vocabulary and site navigation.
C	token	Required by the pathauto module.
S	tree	Provides the Tree (phylogeny) content type.
C	vertical_tabs	Cleans up the node edit page to provide a simplified interface to the publishing options.
C	views	A query builder that allows users to create flexible and dynamic views of node content.
S	view_sort	Allows users to sort content displayed within Taxon pages.
C	weight	Used by the view_sort module.
C	wysiwyg	Enables content to be edited on a site within a WYSIWYG editor.
